# Large scale analysis of protein stability in OMIM disease related human protein variants

**DOI:** 10.1186/s12864-016-2726-y

**Published:** 2016-06-23

**Authors:** Pier Luigi Martelli, Piero Fariselli, Castrense Savojardo, Giulia Babbi, Francesco Aggazio, Rita Casadio

**Affiliations:** Biocomputing Group, University of Bologna, Via San Giacomo 9/2, 40126 Bologna, Italy; Department BiGeA, University of Bologna, Via Selmi 3, 40126 Bologna, Italy; Department BCA, University of Padova, Viale Università 16, 35020 Legnaro (PD), Italy

**Keywords:** Protein stability, Disease related-variations, Residue solvent accessibility, Interactomics networks

## Abstract

**Background:**

Modern genomic techniques allow to associate several Mendelian human diseases to single residue variations in different proteins. Molecular mechanisms explaining the relationship among genotype and phenotype are still under debate. Change of protein stability upon variation appears to assume a particular relevance in annotating whether a single residue substitution can or cannot be associated to a given disease. Thermodynamic properties of human proteins and of their disease related variants are lacking. In the present work, we take advantage of the available three dimensional structure of human proteins for predicting the role of disease related variations on the perturbation of protein stability.

**Results:**

We develop INPS3D, a new predictor based on protein structure for computing the effect of single residue variations on protein stability (ΔΔG), scoring at the state-of-the-art (Pearson’s correlation value of the regression is equal to 0.72 with mean standard error of 1.15 kcal/mol on a blind test set comprising 351 variations in 60 proteins). We then filter 368 OMIM disease related proteins known with atomic resolution (where the three dimensional structure covers at least 70 % of the sequence) with 4717 disease related single residue variations and 685 polymorphisms without clinical consequence. We find that the effect on protein stability of disease related variations is larger than the effect of polymorphisms: in particular, by setting to |1 kcal/mol| the threshold between perturbing and not perturbing variations of the protein stability, about 44 % of disease related variations and 20 % of polymorphisms are predicted with |ΔΔG| > 1 kcal/mol, respectively. A consistent fraction of OMIM disease related variations is however predicted to promote |ΔΔG| ≤ 1 kcal/mol and we focus here on detecting features that can be associated to the thermodynamic property of the protein variant. Our analysis reveals that some 47 % of disease related variations promoting |ΔΔG| ≤ 1 are located in solvent exposed sites of the protein structure. We also find that the increase of the fraction of variations that in proteins are predicted with |ΔΔG| ≤ 1 kcal/mol, partially relates with the increasing number of the protein interacting partners, corroborating the notion that disease related, non-perturbing variations are likely to impair protein-protein interaction (70 % of the disease causing variations, with high accessible surface are indeed predicted in interacting sites). The set of OMIM surface accessible variations with |ΔΔG| ≤ 1 kcal/mol and located in interaction sites are 23 % of the total in 161 proteins. Among these, 43 proteins with some 327 disease causing variations are involved in signalling, structural biological processes, development and differentiation.

**Conclusions:**

We compute the effect of disease causing variations on protein stability with INPS3D, a new state-of-the-art tool for predicting the change in ΔΔG value associated to single residue substitution in protein structures.  The analysis indicates that OMIM disease related variations in proteins promote a much larger effect on protein stability than polymorphisms non-associated to diseases. Disease related variations with a slight effect on protein stability (|ΔΔG| < 1 kcal/mol) frequently occur at the protein accessible surface suggesting that they are located in protein-protein interactions patches in putative human biological functional networks. The hypothesis is corroborated by proving that proteins with many disease related variations that slightly perturb protein stability are on average more connected in the human physical interactome (IntAct) than proteins with variations predicted with |ΔΔG| > 1 kcal/mol.

## Background

One of the key goals in the postgenomic era is the elucidation of the mechanisms at the basis of the relationship between genotype and phenotype. In particular, understanding how human genetic variations are associated to diseases is still an open problem and its solution is a crucial issue for exploiting the possibilities offered by the modern sequencing techniques in the framework of precision medicine [1, 2].

The role of missense mutations inducing single residue variations (SRVs) in proteins has been widely investigated: several databases collect data about the relationship between SRVs and diseases [3] and several predictive tools have been implemented in order to exploit the available knowledge to predict whether new variants are related to diseases ([4–6]; and others listed in [7]) or are affecting protein function [8].

Biophysical studies allowed to measure the thermodynamic effect that protein variations induce on protein stability [9]. However the number of human proteins whose folding thermodynamics is known in the native and mutated form is still limited due to the time consuming and costly procedure at the basis of experimental investigations. To fill the gap, predictive tools have been trained on the available thermodynamic data to compute the free energy change value upon variation ([10–13], and others listed in [14]). Recently, we introduced INPS [15], a sequence based predictor that well compares with tools taking as input protein structure. When dealing with disease related variations in human protein variants, very little is known about their thermodynamics and it is unclear in annotation processes whether a variation perturbing the protein stability is or not disease related. Extensive comparative analyses of the two classes of datasets (phenotypically vs thermodynamically characterized variations) prove that, on average, variation types most involved in disease are also associated to a large effect on protein stability [16–18]. However, the strength of this association, although recently improved (compare results in [16] with [19]), is not sufficient to consider protein destabilization as the only mechanistic cause explaining the insurgence of diseases. Indeed many variations with |ΔΔG| ≤ 1 kcal/mol are disease-related [12, 13, 15, 16, 19]. In this paper, as a follow up to the problem, we specifically deal with OMIM disease related protein variants whose native structure is known and predict the extent of perturbation that the variation may cause on the native protein stability. To this aim, we develop INPS3D, a new tool for computationally estimating the effect of single residue variations on protein stability based on information extracted from protein three dimensional structure, and compare its performance to state-of-the-art predictors on the blind test set of the OMIM related proteins endowed with well resolved structures. By this, we identify a subset of disease-related variations with |ΔΔG| ≤ 1 kcal/mol and prove that these variations often occurs in sites exposed on the protein accessible surface, with a likelihood to be in interaction sites. Integrating these results with human physical interactomic data, we find that on average, proteins endowed with many interaction partners have disease related variations that are solvent exposed and are characterized by low free energy change values. Our results support the hypothesis that, besides protein stability perturbation, impairment of protein-protein interaction can be also a major mechanism explaining the relation between variations and diseases.

## Methods

### Data set

We downloaded from the Humsavar dataset (release 2015_10 of 14 Oct 2015) a collection of 27,185 variations related to 3082 OMIM diseases, on 2367 different human proteins and retained only proteins endowed with a PDB structure (3D) covering at least 70 % of the protein sequence. The PDBSWS resource [20] (August 2015 update) was adopted to map the UniProt sequences onto the PDB structures. We ended up with a dataset of 4717 variations related to 484 OMIM diseases on 368 proteins endowed with PDB structures with resolution lower than 3.0 Å (OMIM set). On the same proteins, we also collected 685 polymorphism lacking evidence of association to disease (POLY set).

To train/test (by adopting a cross validation procedure) the predictors, we used S2648, a dataset that was originally derived from the ProTherm database [9] and corrected by the authors of the PoPMuSiC algorithm [11]. It comprises 2648 variations out of 132 different proteins endowed with a 3D structure. We also evaluated the predictor performances on a blind test of 351 variations in 60 proteins, and on 42 variations of the P53 protein not included in the training set and previously described in [12].

### INPS3D: a structure based method for the prediction of free energy changes upon protein variations

Here we introduce INPS3D that exploits both sequence and structural information to predict the protein stability changes upon single point mutation. INPS3D takes advantage of the recently released INPS [15] that, starting only from protein sequence, performs similarly to the state-of the-art methods based on protein structure. INPS3D is based on nine input features based on protein sequence and structure. The features extracted from protein sequence are, [15]: 1) substitution score derived from the Blosum62 matrix; 2-3) Kyte-Doolittle hydrophobicity scores of native and mutated residues; 4) mutability index of the native residue; 5-6) molecular weights of native and mutated residues; 7) the difference in the alignment score between the native and mutated sequences and an HMM encoding evolutionary information of the target sequence. Two additional real-valued features derived from the protein structures are: 8) the solvent accessibility of the mutated residue, 9) the energy difference between native and mutated proteins. The solvent accessibility is computed with the DSSP method [21] and normalized as previously described [22]. The energy difference is evaluated by using the residue-based contact potential described in [23]. We consider that two residues are in contact if the minimal distance between all the atoms (not including hydrogen atoms) of two residues is ≤ 5 Å. We used the coordinates of the native protein to compute the contact energy and the energy difference as:

1 $$ {\displaystyle \sum_rP\left(r,w\right)}-P\left(r,m\right) $$

where *P* is the contact potential, *w* is the wild-type residue, *m* is the mutated residue, and the *r-*index runs over the list of *w*-neighbouring residues. We tested several other potentials, but the performances were similar or lower than those here reported. INPS3D is based on a Support Vector Regression model (SVR) trained on the same dataset adopted for INPS (see data set section). The adopted conventions on the sign are such as when predicting the ΔΔG associated to a variation, positive values refer to the protein stabilization and negative values to protein destabilization.

### Analysis of protein surfaces

The solvent accessible surface area of residues in wild-type proteins has been evaluated with the DSSP program [21]. In order to obtain the Relative Solvent Accessibility (RSA), solvent accessibility areas were normalized to the residue-specific maximum solvent accessible area, as previously reported [22]. Residues with RSA ≥ 0.2 are classified as accessible, residues with RSA < 0.2 are classified as buried. RSA has been measured on both the protein isolated chain and the protein complex, as downloaded from the repository of “biological assemblies” of the Protein Data Base [http://www.rcsb.org/pdb/download/download.do#Structures]. To define the interaction interface of the complex, we collected the set of residues that are solvent accessible in the isolated chain and are buried in the complex.

### Interactomics analysis

Interacting partners of each protein were retrieved from the IntAct database [24] as downloaded from the IntAct FTP site as to November 2015. The search in the IntAct file was performed using the UniProtKB code and excluding the negative interaction data. The statistical analysis was performed considering only the proteins present in the dataset, at least in one entry.

## Results and discussion

### **INPS3D at work**

INPS3D is a new tool for predicting the change of protein folding free energy induced by single residue variations. The performance of the structure based predictor along with that of the sequence based one [15] are shown in Table 1. We report statistical scores obtained benchmarking the predictors with a more stringent per-protein cross-validation procedure [15] on the S2648 set previously described [11], and on a blind test set including some 351 variations in 60 proteins, and a P53 data set (both not included in the training set). Results, reported in Table 1, indicate that INPS3D outperforms INPS, exploiting structure based features not present in the INPS input encoding. INPS3D well compares with the performances obtained with structure-based state-of-the-art methods, mCSM [12], and MAESTRO, recently made available as web server [13].

**Table 1:** Performance of INPS3D and other state-of-the-art predictors

Method	Cross-validation (2648 variations on 132 proteins)	Blind test set (351 variations on 60 proteins)	Blind test set (42 variations on P53 protein)
INPS^b^	0.53/1.29^a^	0.68/1.26^a^	0.71/1.49^a^
INPS3D	0.58/1.20^a^	0.72/1.15^a^	0.76/1.35^a^
MAESTRO^c^	0.63/1.17^a^	0.71/1.16^a^	0.44/1.71^a,e^
mCSM^d^	0.51/1.26^a^	0.67/1.19^a^	0.68/1.40^a^

^a^Pearson’s correlation coefficient/standard error (kcal/mol)

Data are from ^b^[15]; ^c^[13]; ^d^[12], ^e^this work, respectively

### Predicting the effect of disease related, single residue variations on the stability of OMIM linked proteins

**Fig. 1:**
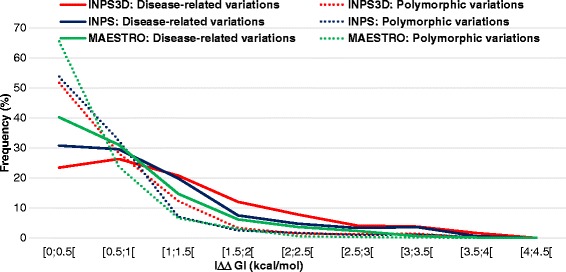
Distribution of the absolute value of the ΔΔG predicted with INPS3D, MAESTRO and INPS. The set includes 4717 disease related variations and 687 polymorphisms in 368 OMIM proteins

We applied INPS (sequence based), INPS3D (structure based) and MAESTRO (structure based) to the OMIM variation set for estimating the change in protein folding free energy induced by the disease-related variations. For sake of comparison we also ran the tools on the POLY set, containing variations not related to diseases, on the same OMIM proteins. We used polymorphisms from the very same proteins that have also variations related to diseases, in order to constrain the ΔG value of the folded form and avoid possible biases due to the inclusion of other proteins. The results (Fig. 1) confirm that disease related variations tend to produce a larger effect on protein stability than polymorphisms, which, on the other hand, appear to promote free energy perturbations mostly distributed within +/-1 kcal/mol. The result is confirmed by all the predictors. INPS3D predicts that 80 % of polymorphisms and 56 % of disease causing variations promote a |ΔΔG| ≤ 1 kcal/mol with respect to the corresponding native protein.

The results are similar with INPS; with Maestro, the fraction of disease-related variations predicted with low |ΔΔG| values increases to 74 % of the total. Our results, obtained with three independent predictors, corroborate the notion that protein stability perturbation (as detected from the predicted |ΔΔG| > 1 kcal/mol) is associated to disease-related variations. However, at least half of the OMIM set is predicted to promote only a slight change in protein stability (within a range of about 1 kcal/mol in absolute value). The observation poses the question as to whether the thermodynamic property of the protein variant (albeit predicted) can be linked to some structural/functional feature of the variation, specifically when it is disease causing. Many investigations addressed the issue of which structural features could be associated to disease related variations ([25–29] and references therein). Conclusions are that genetic variations can have dramatic effects on protein stability, hydrogen bonding networks, conformational dynamics, protein activity and protein interaction networks, particularly at the level of functional assemblies [28]. More recently the correlation between the probability of perturbing the protein stability and that of being disease causing was improved [19] with respect to previous data [16]. However, here our analysis addresses the issue from a different perspective: considering that we have predictors of protein stability, the problem is to which extent they label the overall protein in/stability in relation to the corresponding disease related mutation. We find that a high fraction of the protein variants carrying disease-related mutations are predicted with a low |ΔΔG| value, rather independently of the method (compare the INPS3D to MAESTRO results).

### Protein |ΔΔG| values and structural/functional properties of the variations

In the following we will consider how some structural properties can be clustered considering perturbing and non- perturbing predicted |ΔΔG| values. The analysis focuses on the Relative Surface Accessibility (RSA), on the propensity of the variation to be or not in an interaction patch, and finally on the relation of the protein variant to be in physical interaction with other proteins, considering ΔΔG values predicted with INPS3D.

**Fig. 2:**
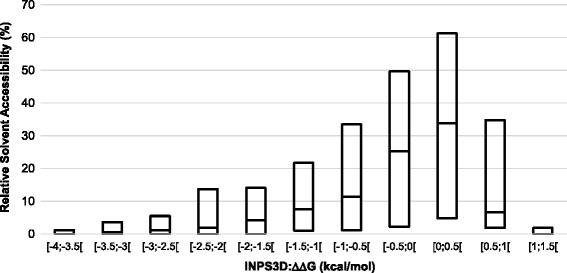
Relative Solvent Accessibility of the variations as a function of ΔΔG predicted for the variants of the OMIM set. The box-plot reports the median and the lower and upper quartiles of the distribution of relative solvent accessibility for each interval of ΔΔG

We analyse the distribution of the relative solvent accessibility (RSA) of the disease related mutations as a function of the free energy change predicted for the corresponding protein variant. Boxplots in Fig. 2 show that the median and the upper quartile values of RSA are higher in the intervals with ΔΔG values close to zero. This indicates that disease related variations with low ΔΔG values have a more spread out distribution of RSA, and then a larger probability to be solvent accessible.

**Fig. 3:**
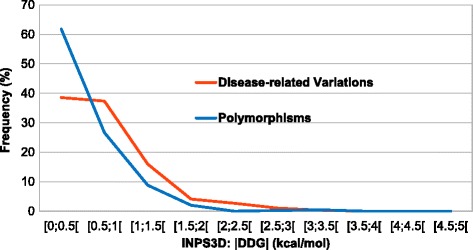
Frequency of the solvent accessible variations as a function of ΔΔG predicted for the protein variants of the OMIM set

In Fig. 3, the distribution of the fraction of solvent accessible variations is plotted as a function of the |ΔΔG| values for disease related and polymorphic protein variants. Low |ΔΔG| values are apparently common both to disease causing and polymorphic variations, when they are located in accessible protein sites.

A detailed grouping of the different behaviour of the structural properties of the OMIM related variations is shown in Tables 2 and 3, as a function of the thermodynamic property of the protein variant. Here we focus also on the difference among monomers and assemblies (as documented in the Protein Data Bank, http://www.rcsb.org/pdb/download/download.do#Structures), in order to highlight the role of protein-protein interactions, when present, in the biological functional unit. As an additional feature, we also included the likelihood of each variations to be or not in an interaction patch (computed with our PRED-PPI, [30]). It appears that disease related mutations in proteins variants with low |ΔΔG| values, when solvent exposed (RSA ≥ 0.20), have also a tendency to be in interaction sites. The property is shared, as expected, with variations that highly perturb protein stability and with polymorphic ones. The low accessibility, in all cases, well agrees with a propensity of being in interaction sites ranging from 0 to 5 %. The value can be considered indicative of the possible range of the false positive rate of the predictor, trained and tested on accessible interaction sites and for which the OMIM set of disease related and polymorphic variations is a blind test set.

**Table 2:** Relation between thermodynamic properties and structural properties in proteins with biologically functional monomeric assembly

Disease-related variant	RSA ≥ 0.20	RSA < 0.20
|ΔΔG| ≤ 1	562 (23.4 %)^a^ *398*	756 (31.4 %)^a^ *39*
|ΔΔG| > 1	176 (7.3 %)^a^ *120*	907 (37.8 %)^a^ *36*
Polymorphic variant		
|ΔΔG| ≤ 1	194 (59.0 %)^a^ *110*	72 (21.9 %)^a^ *3*
|ΔΔG| > 1	22 (6.7 %)^a^ *10*	41 (12.5 %)^a^ *0*

^a^Number of residue predicted to be part of a protein-protein interaction patch (for details on the prediction method, see [30]). Predicted set: 2401 disease related variations and 329 polymorphic variations in 177 proteins

**Table 3:** Relation between thermodynamic properties and structural properties in proteins with biologically functional multimeric assembly

Disease-related variations	RSA ≥ 0.20	RSA < 0.20
|ΔΔG| ≤ 1	660 (28.5 %) Monomer^a^ *465*	650 (28.0 %) Monomer^a^ *24*
	550 (25.0 %) Complex^a^ *421*	760 (31.5 %) Complex^a^ *68*
|ΔΔG| > 1	213 (9.2 %) Monomer^a^ *152*	793 (34.2 %) Monomer^a^ *24*
	196 (8.5 %) Complex^a^ *140*	810 (35.0 %) Complex^a^ *36*
Polymorphic variations		
|ΔΔG| ≤ 1	198 (55.6 %) Monomer^a^ *131*	84 (23.6 %) Monomer^a^ *5*
	186 (52.2 %) Complex^a^ *119*	96 (27.0 %) Complex^a^ *17*
|ΔΔG| > 1	29 (8.1 %) Monomer^a^ *21*	45 (12.6 %) Monomer^a^ *9*
	29 (8.1 %) Complex^a^ *21*	45 (12.6 %) Complex^a^ *9*

^a^Number of residue predicted to be part of a protein-protein interaction patch. 2316 disease related variations and 356 polymorphic variations in 191 proteins. Predictions of INPS-3D and PRED-PPI are independent of the assembly state. RSA values were independently estimated on the monomeric and the complex structures

Distinguishing functional monomeric from multimeric biological assemblies highlights the relevance of the variations when they are located at the interface of protein complexes [28]. In Table 3, the same grouping of Table 2 is therefore shown for proteins with a biologically functional assembly, as documented in the PDB. Here, it appears that only a small fractions of the total number of disease related mutations in the set occurs at the monomer interface (compare Monomer and Complex at RSA ≥ 0.20) and concomitantly also the number of interaction sites predicted on the complex interface is very low.

From the data reported in Tables 2 and 3, it can be computed that about 70 % of the disease causing variations with high accessible surface in monomers are predicted to be part of an interaction patch. The result is particularly significant considering that the fraction of all accessible residues predicted in interaction patches on the same 368 proteins is 55 %.

Summing up, we show that disease related variations in proteins can promote a low |ΔΔG| value, particularly when they are located in accessible sites that are also interacting sites.

**Fig. 4:**
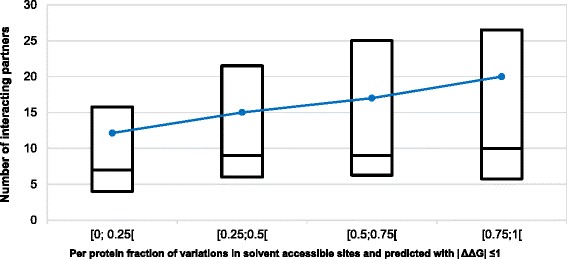
Relation between the per-protein fraction of non-perturbing, solvent accessible variations and the corresponding number of the wild-type partners of interactions in the human interactome. The box-plot reports the median and the lower and upper quartiles of the number of interactions present in IntAct as a function of the fraction of solvent accessible, non-perturbing variations. The dashed blue line connects the average values. Non perturbing variations are those predicted to promote a |ΔΔG| ≤ 1 kcal/mol with INPS3D and found in protein sites that are solvent accessible. Data refers to 170 proteins with 4037 variations of our data set. Proteins with less than 5 disease-related variations or without interactomic data reported in IntAct are excluded

**Fig. 5:**
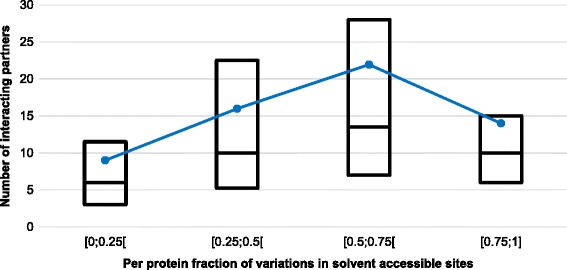
Relation between the per-protein fraction of solvent accessible variations and the corresponding number of the wild-type partners of interactions in the human interactome. The box-plot reports the median and the lower and upper quartiles of the number of interactions present in IntAct as a function of the fraction of solvent accessible variations. The dashed blue line connects the average values. Data refers to 170 proteins with 4037 variations of our data set. Proteins with less than 5 disease-related variations or without interactomic data reported in IntAct are excluded

As a follow up, one may consider to which extent protein variants with disease-related mutations located in solvent exposed sites and slightly perturbing the stability, are or not involved in interaction networks of physical interaction, as available in IntAct [24]. We collected from IntAct the number of interacting partners for each protein and analysed it as a function of the fraction of solvent accessible, non-perturbing variations (Fig. 4). The upper quartile and the mean values of the number of interacting partners per protein increase as the fraction of disease related variations predicted as non-perturbing increases. When all the solvent exposed disease related mutations (RSA ≥ 20 %) per protein are related to the number of the corresponding protein interacting partners (Fig. 5), the trend is different from that observed in Fig. 4. This observation highlights the role of predicted ΔΔG values for determining the relation among protein variants with disease-related mutations located in solvent exposed sites and slightly perturbing the stability, and the number of interacting partners in a protein-protein interaction network.

The proteins endowed with a large amount of non-perturbing and solvent exposed disease related variations seem to play a central role in the human protein-protein interaction network. Likely, a variation on the protein surface can affect the interaction affinity, affecting important biological pathways and leading to an altered phenotype, as recently described [31]. Out of the 43 proteins for which at least 50 % of disease related variations are solvent exposed and predicted with |ΔΔG| ≤ 1, 42 % are involved in differentiation and development processes (including insulin, calmodulin, noggin, angiogenin), 40 % are involved in signalling processes (including the GTPases KRAS, HRAS and NRAS, the serine/threonine kinases PIK3CA and CHEK2), 23 % are structural and adhesion proteins (e.g., actins ACTA1, ACTG2, tubulin TUBA1A and integrin β2).

## Conclusions

We address the problem of the perturbations of the protein stability by disease causing variations on a set of OMIM related proteins whose native structure is well solved. To this aim we implemented INPS3D, a tool for computationally estimating the change in ΔΔG value associated to single residue variations, taking as input protein structure. Our strategy is to adopt a predictor that scores at the state-of-the-art and we compare its performance to other state-of-the-art predictors. INPS3D exploits information extracted from protein structures and outperforms the recently released INPS, based only on sequence information. Moreover INPS3D outperforms state-of-the-art structure-based methods that perform similarly to INPS and well compares with MAESTRO, which recently became available as a web server [13]. Both predictors agree up to 90 % even in regions of |ΔΔG| values that can be considered below the error limit of the predictors. We found that OMIM disease-related variations in proteins generally promote a much larger effect on protein stability than polymorphisms non-associated to diseases on the same proteins, confirming that stability perturbation plays a crucial role in impairing protein function (recently confirmed also in [31]). Nevertheless, a significant fraction of disease related variations is predicted to have a small perturbation effect on protein stability: about 50 % of variations promote a |ΔΔG| <1 kcal/mol. The structural analysis of the corresponding proteins reveals that disease-related variations with a slight effect on protein stability often occur on the protein surface suggesting that they can affect the interaction of the proteins within biological functional networks. The analysis of protein-protein interaction networks corroborates the hypothesis that proteins with many non-perturbing disease-related variations are more connected in the human physical interactome (IntAct) than proteins with variations predicted with |ΔΔG| > 1 kcal/mol. The results are however indicative. The error associated to the computed |ΔΔG| value by our predictors (Table 1) is competing with the range of small changes in protein stability and this could increase the number of variations actually destabilising protein stability. It should also be mentioned that for each protein other features that are not exploited in this analysis (e.g., solubility, post-translational modifications, subcellular location, level of expression, etc.) may be considered when labelling a variations as disease causing.

## Abbreviations

RSA: relative solvent accessibility
